# Twenty-four hour urinary sodium and potassium excretion in adult population of Slovenia: results of the Manjsoli.si/2022 study

**DOI:** 10.1017/S1368980024001605

**Published:** 2024-09-16

**Authors:** Saša Kugler, Urška Blaznik, Maruša Rehberger, Metka Zaletel, Aleš Korošec, Matej Somrak, Adrijana Oblak, Igor Pravst, Maša Hribar, Anita Kušar, Jana Brguljan-Hitij, Simona Gaberšček, Katja Zaletel, Ivan Eržen

**Affiliations:** 1 University of Ljubljana, Faculty of Medicine, Ljubljana, Slovenia; 2 National Institute of Public Health, Ljubljana, Slovenia; 3 University Medical Centre Ljubljana, Ljubljana, Slovenia; 4 Nutritional Institute, Ljubljana, Slovenia

**Keywords:** 24-h urine, Sodium, Potassium, Salt intake, Potassium intake

## Abstract

**Objective:**

The objective of study was to assess 24-h urinary Na and K excretion and estimate the average salt and K intakes in a nationally representative sample of the adult population of Slovenia.

**Design:**

A nationally representative cross-sectional study was conducted in four stages between September and November 2022: study questionnaire, physical measurements, 24-h urine collection and laboratory analysis.

**Setting:**

Slovenia.

**Participants:**

We invited 2000 adult, non-institutionalised inhabitants of Slovenia, aged between 25 and 64 years. A stratified two-staged sample was selected from this population by the Statistical Office of Slovenia, using sampling from the Central Population Register. According to the WHO methodology, additional eligibility criteria were screened before participating. A total of 518 individuals participated in all four stages of the study, resulting in a response rate of 30 %.

**Results:**

The mean 24-h urinary Na excretion was 168 mmol/d (95 % CI 156, 180), which corresponds to a mean estimated intake of 10·3 g salt/d (95 % CI 9·6, 11·1). Mean 24-h urinary K excretion was 65·4 mmol/d (95 % CI 63·2, 67·5), and the estimated mean K intake was 2·93 g/d (95 % CI 2·84, 3·03). There were statistically significant differences in mean intakes between males and females. The mean sodium-to-potassium ratio was 2·7 (95 % CI 2·5, 2·8).

**Conclusions:**

The study results highlighted that the salt intake in the adult population of Slovenia remains much higher than recommended by the WHO, and K intakes are insufficient, as most participants did not meet the recommendations.

High Na intake is one of the most important diet-related factors for developing high blood pressure (BP), which is in turn among the leading risk factors attributable to deaths globally^(1)^. In 2019, a total number of 1·72 million deaths and 40·5 million disability-adjusted life years were caused by CVD due to high dietary intakes of Na globally^(2)^. Earlier results also suggest that globally 1·65 million annual deaths from cardiovascular causes were attributable to Na intakes above the recommended level^(3)^. Systematic reviews and meta-analyses have shown a reduction in BP with lower intakes of Na, both in normotensive and hypertensive individuals^(4–6)^.

To reduce the risks of high BP and CVD, the WHO has therefore recommended that the daily Na intake should be no more than 2·0 g/d (approximately 5·0 g salt/d)^(7)^. Processed foods and meals (e.g. ready-made meals, salty snacks, bread and meat products, and restaurant-prepared foods) contribute the most Na to the diet. In European countries and Northern America, they account for more than 75 % of daily Na intake^(8,9)^. In most countries of the WHO European region, the average Na/salt intakes are above the recommended level^(10)^; in fact, it appears that most countries worldwide do not meet the recommendations^(11)^.

Another important dietary component in the prevention of high BP is K. Results of systematic reviews and meta-analyses have shown that a higher K intake lowers the risk of high BP^(12,13)^; higher K intake was also associated with a decreased risk of stroke^(14)^. To reduce the risk for the development of high BP and better management of such condition, the WHO has recommended increasing dietary K intake to at least 90 mmol/d (or 3·5 g/d)^(15)^. Recent estimates of K intake in Slovenia, based on 24-h urinary excretion, suggest insufficient K intake; however, these results were from a smaller sample of subjects, living in elderly care institutions^(16)^.

Previous estimates of salt intake based on 24-h urinary excretion in Slovenian adults were done approximately 10 years ago; estimates of K intake in adults are also lacking. Therefore, the main objective of our study was to assess 24-h urinary Na and K excretion and estimate the average salt and K intakes in a nationally representative sample of 25–64-year-old adult population of Slovenia.

## Methodology

### Sample and data collection

The ManjSoli.si study was based on the WHO methodology^(17)^. Given the sensitive nature of the data collected, the study was confidential in each stage and anonymised in some stages. Therefore, each invited individual had their own two identification numbers for different stages of study; the first one was represented by a numerical value, while the other was concealed within a barcode. To assure that individuals could not be recognised from any given data, only authorised personnel at the National Institute of Public Health (NIJZ) had the key for data linking.

The ManjSoli.si cross-sectional study was conducted between 14 September and 10 November 2022, among 2000 adult, non-institutionalised inhabitants aged between 25 and 64 years. A stratified two-staged sample was selected from this target population by the Statistical Office of Slovenia, using sampling from the Central Population Register in accordance with the National Statistics Act^(18)^. The sample size was calculated as described in the WHO protocol^(17)^. To detect approximately 1 g reduction in salt intake over time using 24-h urinary Na excretion, with sd of 3 g salt/d (α = 0·05, power = 0·80), a minimum sample of 141 individuals per stratum is recommended. We initially stratified the population by sex (male and female) and age groups (25–44 and 45–64 years) which results in 564 individuals. Considering expected response rate of about 30 % and our expert recommendations, the final sample size was rounded to 2000 individuals. The sample was stratified explicitly according to the size and type of settlement and implicitly according to the statistical regions (Nomenclature of Territorial Units for Statistics (NUTS) – 3)^(19)^. The first-stage units were chosen using probability proportional to size based on the number of residents aged between 25 and 64·99 years. Each sampling point consisted of ten individuals (first-stage units). A total of 200 sampling points were selected across Slovenia, proportional to the number of residents in each region. Selected individuals from all twelve statistical regions (NUTS – 3) had to be 25 years or older as of 1 September 2022 and not older than 65 years as of 1 December 2022.

**Fig. 1:**
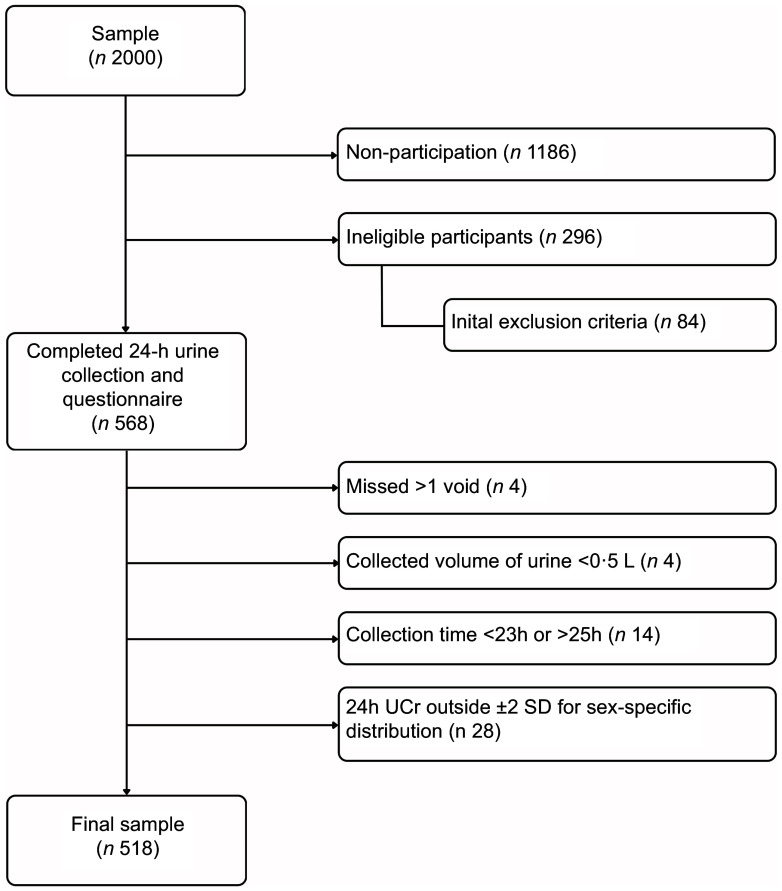
Flow chart of exclusion process in the Manjsoli.si study

To accommodate the complex stages of the study, Computer-Assisted Personal Interviewing (CAPI) mode was exclusively implemented through outsourcing. The exclusion process and final sample size are presented in Fig. 1. The response rate was 30 %.

At least 3 d prior, each participant received an introductory letter via postal mail, which informed them of the study and the arrival of a field researcher. Each participant was visited in person. In the case of a failed initial contact, the field researcher contacted the participant at least three additional times, on different days of the week and at different times of the day. In the ManjSoli.si study, twenty-one well-trained field researchers collaborated. All field researchers had prior experience and attended a 4-h educational workshop on the study protocol.

During the initial visit by the field researchers, participants were introduced to the study protocol and screened for eligibility to participate in the study, based on initial exclusion criteria. Before inclusion in the study, all participants were informed of their right to voluntarily participate in the study and to withdraw at any time. Those who agreed to participate signed the informed consent form (ICF) in duplicate.

For individuals to have successfully participated in the study, ICF had to be signed in two copies and all four stages of the study completed (1) a study questionnaire, (2) physical measurements, (3) 24-h urine collection and (4) laboratory analysis (explained below). Field researchers were responsible for participants completing the initial three stages of the study, enabling the laboratory to perform the analyses (stage four). Participants who successfully completed all stages of the study were also awarded with practical gifts and results of the laboratory analysis.

### Study questionnaire

The study questionnaire was designed according to the WHO protocol^(17)^, with added questions from other national surveys. The questionnaire contained forty-two questions and was divided into separate sections for initial exclusion criteria, sociodemographic data (age, education and cohesion region) and other. Data on education were classified by International Standard Classification of Education (ISCED)^(20)^ and grouped into three categories: (a) primary or less (ISCED level 1–2); (b) secondary (ISCED level 3–4) and (c) tertiary or more (ISCED level 5–8). For data on cohesion regions NUTS – 2, classification system was used^(19)^.

### Anthropometric, blood pressure and heart rate measurements

After the completed questionnaire, participants were asked to relax for further measurements. During the anthropometric measurements, the participants were in light clothing and without shoes. The protocol for measurements of height and weight was adapted from the Slovenian national food consumption survey – SI.Menu^(21)^. Body height was measured to the nearest 0·5 cm using a wooden corner block and non-stretch measuring tape (SECA 201, Seca) with the participants’ head in Frankfort horizontal plane. Body weight was measured to the nearest 0·1 kg using calibrated digital scales (Tanita BC-730). The measurements were done preferably at the first visit.

Each participant’s BP and heart rate were measured in a sitting position, with their back supported and legs uncrossed, using validated measuring device OMRON M7 Intelli IT (Omron Co). The measurements were done primarily on the left arm and repeated three times, with 1-min breaks between each measure. The average of the last two measurements was used for the final analysis. Based on these measurements, participants were grouped according to ESC/ESH guidelines for the management of arterial hypertension^(22)^.

### 24-h urine collection

After signing ICF and completing the previous two stages of the study, participants received a 3-litre plastic container (Vacuette Uri-Plus, Greiner Bio-One), together with detailed written and oral instructions for the collection of 24-h urine. Both the container and the signed ICF were equipped with individual labels containing the participant’s barcode. The container was additionally equipped with an empty label for writing down the starting and ending times of urine collection and potentially missed voids.

On the collection day, participants would discard the first void and write down the starting time. They collected urine until approximately the same time next day. Para-aminobenzoic acid was not used. During the collection period, the samples were stored in the coldest part of the house. Within 24 h after the end of the collection, the samples were collected from participants and transferred them to the Institute of Clinical Chemistry and Biochemistry at the University Medical Centre in Ljubljana (ICCB, UMCL). There the collected volume of samples was recorded. Afterwards, samples were thoroughly mixed and aliquoted into samples of 10 ml, which were then stored at –20 °C until analysis. The barcode on all received materials ensured that the laboratory could verify whether the participant who collected the 24-h urine sample had given their consent for the study. Additionally, the barcode facilitated seamless analysis of the participant’s 24-h sample by the laboratory.

### Laboratory analysis

Analysis of Na and K concentration in urine was performed using indirect potentiometry with ion-selective electrodes on biochemical analyser Abbott Alinity c (Abbott) and expressed as mmol/l. Creatinine concentration was determined using an enzymatic reaction followed by spectrophotometric detection, also on biochemical analyser Abbott Alinity c (Abbott). The result was expressed as mmol/l.

### Exclusion criteria

At the beginning of the interview, each participant was screened for the following initial exclusion criteria (adapted from WHO^(17)^ and supplemented): (a) unable to provide informed consent, (b) known history of heart or kidney failure, stroke or liver disease, (c) pregnancy, (d) recently began or changed therapy with diuretics (less than 2 weeks), (e) recently began or changed high BP therapy (less than two weeks) and (f) other conditions that would make 24-h urine collection difficult.

For quality control of the returned urine samples, the following exclusion criteria were applied: (a) volume of urine is <500 ml; (b) participant reported more than one missed void; (c) the time of urine collection was <23 h or >25 h; (d) the time from the end of collection to delivery to laboratory was >24 h and (e) the 24-h urinary creatinine concentration was outside ± 2 s d for sex-specific distribution.

### Statistical analysis

The study results were first analysed using descriptive statistics. Numerical variables are expressed as means and 95 % CI. Categorical variables are expressed as percentages. To estimate dietary intakes of Na and K, we first converted urinary excretions of Na and K (in mmol/d) into mg/d (1 mmol Na = 23 mg Na; 1 mmol K = 39 mg K). We then multiplied Na intake by 2·542 to estimate salt intake. To account for non-urinary losses, we also multiplied Na values by a factor of 1·05 (assuming approximately 95 % of ingested Na is excreted in the urine)^(17)^. Similarly, K values were multiplied by 1·15, assuming approximately 85 % of ingested K is excreted in the urine^(17)^. The urinary sodium-to-potassium (Na:K) ratio was determined by dividing urinary excretions of Na and K (in mmol/d) for each participant. A multivariable linear regression analysis was also conducted to examine the relationship between measured systolic blood pressure (SBP) and diastolic blood pressure (DBP) with urinary Na and K excretion (in mmol/d) and adjusted for BMI (kg/m^2^), age (years) and sex (female – 0, male – 1).

The χ^2^ was used to investigate associations with sociodemographic and other explanatory variables. In addition, the Bonferroni correction was used as a method to determine the statistical difference between studied groups. Differences between numerical variables were analysed using ANOVA. The level of statistical significance was set at *P* < 0·05. The article largely presents data where the se of the share estimate was 5 % or less, meaning that the estimate was sufficiently accurate^(23)^.

Data presented in the article were weighted by sex, age group, education, cohesion region and combinations (sex*age and sex*cohesion region). To reduce the impact of non-response bias, the weighting was conducted for the entire population using the raking method with the reference date of 1 January 2022. The comparison between the sociodemographic data of the initial sample and the final weighted sample shows no more than about 2 % points difference and is presented in see online supplementary material, Supplementary Table 1. The minimum of weights used was 0·37 and maximum 3·94.

The statistical analysis was conducted using SPSS, version 25 (IBM, 2022).

## Results

### Characteristics of the participants

Five hundred and sixty-eight participants finished all four stages of the study, out of which fifty were excluded due to incomplete 24-h urine samples as determined by collected volume, number of missed voids, time of urine collection and 24-h urinary creatinine concentration. Five hundred and eighteen participants (52 % males and 48 % females) were included in the final analysis. Characteristics of the participants are presented in Table 1. Males had significantly higher average BMI than females (27·9 *v*. 26·3, respectively). Overall, 35 % of participants had optimal BP measurement, with statistically significant differences between sexes (19 % of males *v*. 52 % of females).

**Table 1:** Age, education, cohesion region, BMI and blood pressure measurements in a sample of the adult population of Slovenia

	Total (*n* 518)				Males (*n* 227)				Females (*n* 291)				
	Mean	95 % CI	%	*n*	Mean	95 % CI	%	*n*	Mean	95 % CI	%	*n*	*P*-value
Age (years)	45·4	44·5, 46·4			45·2	43·9, 46·6			45·7	44·3, 47·1			0·664
Age groups (years)													0·955
– 25–34			21	109			22	58			20	51	
– 35–44			27	138			27	73			26	65	
– 45–54			26	137			26	71			26	66	
– 55–64			26	134			25	67			22	67	
Education													0·034
– Primary or less			12	63			11	31			13	33	
– Secondary			55	285			60	163			49	122	
– Tertiary or more			33	169			28	76			38	93	
Cohesion region													0·835
– West			53	274			53	144			52	130	
– East			47	244			47	126			48	118	
BMI (kg/m^2)^	27·1	26·6, 27·6			27·9	27·2, 28·6			26·2	25·6, 26·9			<0·001
BMI categorisation													<0·001
– Underweight (<18 kg/m^2^)			1·2	6			0·8	2			1·7	4	
– Normal (18·5–24·9 kg/m^2^)			37	190			27	74			47	116	
– Overweight (25–29·9 kg/m^2^)			36	186			42	113			30	74	
– Obese (>30 kg/m^2^)			26	135			30	81			22	54	
SBP (mmHg)	124	122, 126			130	128, 132			117	115, 120			<0·001
DBP (mmHg)	80·9	79·9, 81·9			82·9	81·5, 84·3			78·8	77·4, 80·1			<0·001
BP measurement categorisation													<0·001
– <120 SBP and <80 DBP (mmHg)			35	176			19	41			52	135	
– 120–129 SBP and/or 80–84 DBP (mmHg)			22	113			27	59			17	54	
– 130–139 SBP and/or 85–89 DBP (mmHg)			17	94			22	53			12	41	
– 140–159 SBP and/or 90–99 DBP (mmHg)			19	99			22	54			14	45	
– 160–179 SBP and/or100–109 DBP (mmHg)			6·4	32			8·3	17			4·3	15	
– ≥180 SBP and/or ≥110 DBP (mmHg)			0·7	4			1·3	3			0·2	1	

BP, blood pressure; SBP, systolic blood pressure; DBP, diastolic blood pressure.

*P*-value <0·05 was considered as statistically significant.

### Urinary sodium and potassium excretions, and estimated intakes

The results of laboratory analyses are presented in Table 2. Males had significantly higher urinary creatinine excretion than females (*P* < 0·001). They also had higher urinary Na and K excretion than females (142 *v*. 191 mmol/d and 60·7 *v*. 69·7 mmol/d, respectively), which can also be observed in the mean estimated intakes.

**Table 2:** Urinary volume, urinary creatinine, Na and K excretions, estimated intakes and molar ratio of Na and K in a sample of the adult population of Slovenia

	Total (*n* 518)		Males (*n* 227)		Females (*n* 291)		
	Mean	95 % CI	Mean	95 % CI	Mean	95 % CI	*P*-value
Volume (L)	1·95	1·9, 2·0	2·02	1·9, 2·1	1·88	1·8, 2·0	0·0408
Urinary creatinine excretion (mmol/d)	12·3	11·8, 12·7	14·7	14·1, 15·3	9·62	9·3, 9·9	<0·001
Urinary Na excretion (mmol/d)	168	156, 180	191	175, 206	142	124, 160	<0·001
Urinary K excretion (mmol/d)	65·4	63·2, 67·5	69·7	66·6, 72·9	60·7	57·8, 63·5	<0·001
Estimated salt intake (g/d)	10·3	9·6, 11·1	11·7	10·7, 12·7	8·72	7·6, 9·8	<0·001
Estimated K intake (g/d)	2·93	2·84, 3·03	3·13	2·99, 3·27	2·72	2·59, 2·84	<0·001
Sodium-to-potassium ratio (mmol/mmol)	2·66	2·54, 2·76	2·82	2·68, 2·96	2·48	2·32, 2·63	<0·001

*P*-value <0·05 was considered as statistically significant.

The mean estimated salt intake among participants with measured 140–159 SBP and/or 90–99 DBP mmHg was 11·9 g/d (95 % CI 10·7, 13·0), which was significantly higher compared with participants with measured optimal (<120 SBP and <80 DBP mmHg) BP (8·9 g/d (95 % CI 8·3, 9·5); *P* < 0·001). A significant difference was also observed compared with participants with measured normal (120–129 SBP and/or 80–84 DBP mmHg) BP (9·8 g/d (95 % CI 9·0, 10·5); *P* < 0·05) (see online supplementary material, Supplementary Table 2). Overweight participants had significantly higher mean salt intake compared with normal-weight participants (10·7 g/d (95 % CI 9·93, 11·4) *v*. 8·41 g/d (95 % CI 7·85, 8·95), respectively; *P* < 0·001). Similarly, obese participants had a significantly higher mean salt intake (11·7 g/d (95 % CI 10·8, 12·6)) than normal-weight participants (p < 0·001) (see online supplementary material, Supplementary Table 3).

A multivariable linear regression analysis was conducted to test if urinary Na and K excretion predicted SBP and DBP.

Overall, unadjusted regression model with SBP as dependent variable was statistically significant (R^2^_adj_ = 0·05, F(2; 263)=8·01; *P* < 0·001). It was found that urinary Na excretion significantly predicted SBP (*B* = 0·05, 95 % CI (0·02, 0·08), *P* = 0·001), but urinary K excretion was not a significant predictor (*B* = 0·02, 95 % CI (–0·07, 0·11), *P* = 0·696). Results of unadjusted regression model with DBP were somewhat similar. The overall regression was significant (R^2^_adj_ = 0·02, F(2; 263)=3·22; *P* = 0·042), with urinary Na excretion as a significant predictor (*B* = 0·02, 95 % CI (0·002, 0·038), *P* = 0·026). Urinary K excretion was also not significant (*B* = 0·004, 95 % CI (–0·05, 0·06), *P* = 0·894).

The results of models adjusted for BMI, age and sex are presented in Table 3. The overall regression with SBP as dependent variable was statistically significant (R^2^_adj_ = 0·29, F(5; 260)=22·8; *P* < 0·001). Results show urinary Na excretion significantly predicted SBP (*B* = 0·035; *P* = 0·013). It was also found that urinary K excretion had a negative association with SBP; however, it was not statistically significant (B = –0·027; *P* = 0·495). The overall regression with DBP was also statistically significant (R^2^_adj_ = 0·14, F(5; 260)=9·55; *P* < 0·001). Results showed that neither urinary Na excretion nor urinary K excretion significantly predicted DBP (Table 3).

**Table 3:** Results of multivariable linear regression analysis with SBP and DBP as dependent variables in a sample of adult population of Slovenia

	SBP (mmHg)			DBP (mmHg)		
	*B*	95 % CI for *B*	*P*-value	*B*	95 % CI for *B*	*P*-value
Constant	78·4	66·6, 90·1	<0·001	57·5	50·0, 64·9	<0·001
Urinary Na excretion (mmol/d)	0·035	0·01, 0·06	0·013	0·014	–0·003, 0·032	0·114
Urinary K excretion (mmol/d)	−0·027	–0·11, 0·05	0·495	−0·018	–0·07, 0·03	0·483
BMI (kg/m^2^)	0·35	–0·05, 0·76	0·087	0·45	0·19, 0·71	0·001
Age (years)	0·61	0·44, 0·79	<0·001	0·22	0·11, 0·33	<0·001
Sex (female = ref.)	9·73	5·79, 13·7	<0·001	1·29	–1·19, 3·77	0·308
R^2^	0·31			0·16		
R^2^_adj_	0·29			0·14		

SBP, systolic blood pressure; DBP, diastolic blood pressure.

*P*-value <0·05 was considered as statistically significant.

**Fig. 2:**
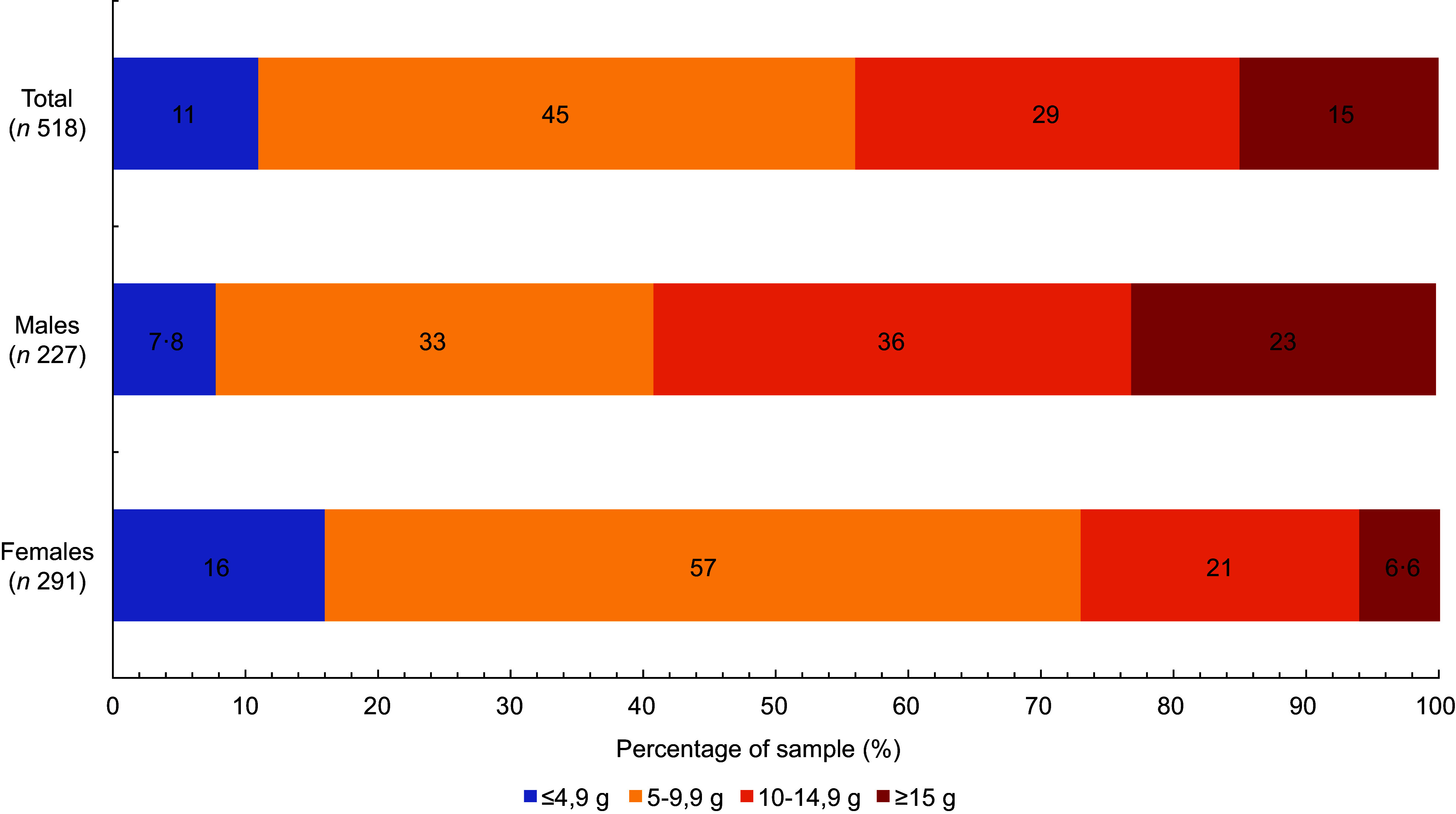
Percentage of individuals meeting the WHO recommendation for salt intake (<5 g/d) or exceeding it, in a sample of adult population of Slovenia, by sex

Only 11·5 % of participants had salt intakes below 5·0 g salt/d, as recommended by WHO (8) (Fig. 2). There were also statistically significant differences between sexes, with 7·8% of males and 15·5% of females having estimated intakes below 5 g/d (*P* < 0·001).

**Fig. 3:**
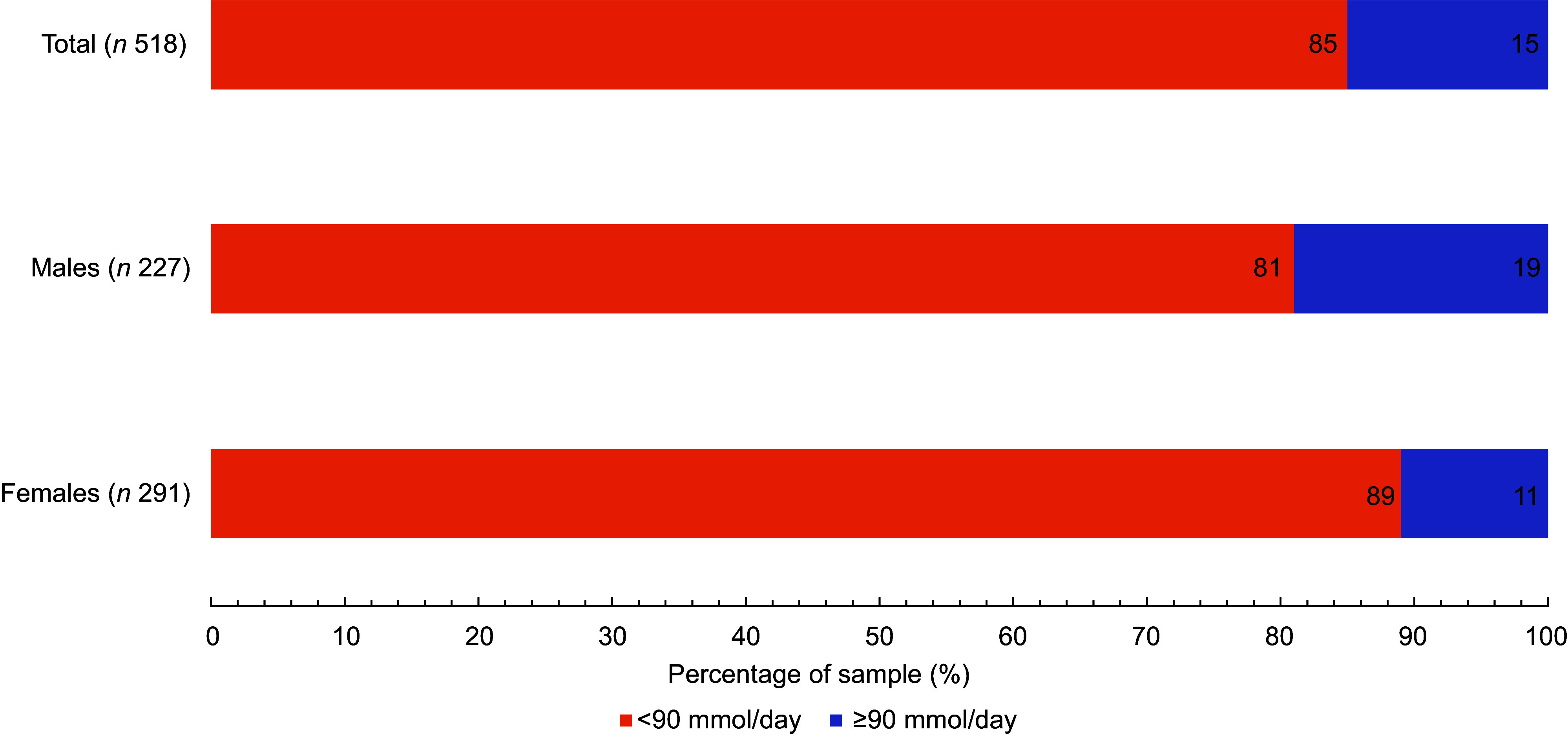
Percentage of adults meeting the minimal daily K intake as recommended by the WHO (≥90 mmol/d or ≥3·5 g/d) in a sample of the adult population of Slovenia, by sex

Similarly, only 15 % of participants (19 % of males and 11 % of females) met the recommended intake of 90 mmol K per d (3·5 g/d) or more^(15)^ (Fig. 3). Furthermore, 3·9 % of participants met the recommended Na:K ratio of about 1 or lower (3·0 % of males and 4·9 % of females; *P* = 0·268)^(15)^.

## Discussion

Results of our study show that the estimated average salt intake in the adult population of Slovenia is above the recommended level, and K intake is insufficient.

The mean urinary Na excretion in our sample of the adult population of Slovenia was 168 mmol/d, which corresponds to an estimated average salt intake of 10·3 g/d, twice the amount recommended by the WHO. However, when compared with previous estimates done in the adult population of Slovenia in 2010 (12·4 g/d)^(24)^, the results suggest a slight improvement in the last decade. Nevertheless, only 11·5 % of participants met the WHO recommended intake of less than 5 g salt/d^(7)^. Our results are consistent with other recent studies, for example in Lithuania, where results show an estimated average salt intake of 10·0 g/d, with males having a significantly higher intake than females (11·7 g/d *v*. 8·4 g/d, respectively)^(25)^. In Italy, urinary Na excretion in adults was 189 mmol/d for males and 147 mmol/d for females, which corresponds to estimated salt intakes of 10·9 g/d and 8·5 g/d, respectively^(26)^. Results from a study in Podgorica, Montenegro, also show a high average salt intake (11·6 g/d), with only 7 % of participants meeting the recommended intake^(27)^. Similar trends can be observed in other countries and regions; in a systematic review of global, regional and national Na intakes by Powles *et al.*, it was estimated that the global average salt intake was 10·06 g/d (95 % CI 9·88, 10·21 g/d), with intakes being highest in East Asia, Central Asia and Eastern Europe^(11)^. It has been estimated that 95–99 % of the adult population in the world had intakes of Na (salt) above the recommended limits^(3)^; therefore, our results were not surprising. There were significant differences between sexes in urinary Na excretions and corresponding estimated intakes, which was to be expected as Na/salt intake is measured as total intake and not adjusted for energy intake or body mass^(10)^.

Our study also indicates an insufficient K intake in the adult population of Slovenia. The average K excretion was 65·4 mmol/d, which corresponds to 2·93 g of K per d. Only 15 % of participants met the minimum daily intake of ≥90 mmol/d (or ≥3·5 g/d) as recommended by the WHO^(15)^. In Lithuania, slightly higher urinary K excretion was observed (73·8 ± 29·6 mmol/d) and estimated K intake was 3·3 ± 1·3 g/d; approximately 23·1 % of participants were meeting the recommendations^(25)^. Studies from Northern Greece and in the Sultanate of Oman also showed insufficient intakes among adults, with average K excretions of 65·1 ± 24·6 mmol/d and 52·6 ± 32·6 mmol/d (3·3 ± 1·25 and 2·36 ± 1·46 g/d, respectively)^(28,29)^. A recent systematic review on global K intake determined the global mean K intake at 2·25 g/d (95 % CI 2·05, 2·44 g/d), with the highest intakes in Eastern (3·53g/d, 95 % CI 3·05, 4·01 g/d) and Western Europe (3·29 g/d, 95 % CI 3·13, 3·47 g/d)^(30)^. We found significant differences between sexes, with males having a higher intake than females. This may be due to the same reasons as with salt intake.

The results of the multivariable regression analysis show that urinary Na excretion is a significant predictor of SBP (*B* = 0·035, but not of DBP, after adjusting for sex, BMI and age. The coefficient (B) for urinary Na excretion was 0·035, which indicates that with each mmol/d increase in urinary Na excretion, SBP would increase by 0·035 mmHg. Similar results were found in the INTERSALT study. Their results showed that for every 10 mmol increase in urinary Na excretion, SBP increased by 0·217 mmHg^(31)^. Although there was a negative association between urinary K excretion and SBP or DBP after adjustment, this was not statistically significant. A similar finding was reported in a study on adults from Malaysia^(32)^. In addition, the R^2^_adj_ was 0·29 for adjusted regression with SBP as dependent variable, meaning that the included independent variables explained only 29 % of the variance. In the adjusted regression with DBP as dependent variable, the R^2^_adj_ was even lower at 0·14. This is likely due to either not measuring or not including all the factors which can influence SBP or DBP.

High Na and lower K intakes are reflected in the urinary Na:K ratio; in our study, the mean ratio was 2·7. While no formal reference values are set, the WHO considers the optimal Na:K ratio to be approximately one-to-one^(15)^; our results therefore show less of than optimal situation. A study from Italy, which collected data from a sample of adults from two time periods, showed that the mean ratio between 2018 and 2019 was 2·9 in men and 2·6 in women^(33)^, which is in line with our results. Similar results were also found in Northern Greece^(28)^. A slightly lower ratio was observed in Lithuania, where the mean Na:K ratio was 2·3 (sd 1·1), with a significant difference between sexes (2·6 in males *v*. 2·1 in females). Additionally, their results also show a higher percentage of participants with Na:K ratio about 1 or lower, compared with our results^(25)^.

There is consistent evidence that a reduction in Na/salt intake is associated with a decrease in BP, both in hypertensive and normotensive subjects^(4,6)^. A similar association with BP has also been identified in K, as higher intakes could lead to a reduction of risk for developing high BP^(13)^ or stroke^(14)^. Studies have also suggested that the ratio of Na to K may have stronger associations with SBP and CVD risk than either Na or K alone^(31,34)^. For example, the results of the INTERSALT study showed a significant association between the Na:K ratio and SBP. Furthermore, it was estimated that by lowering the ratio from 3·09 to 1·00, the population average SBP would be lower by 3·36 mmHg^(31)^. A study by Cook et al observed a significant linear association between the Na:K ratio and the risk of CVD^(34)^. Similarly, Ma *et al.* reported a 24 % increase in cardiovascular risk for each unit increase in Na:K ratio (hazard ratio 1·24; 95 % CI 1·12, 1·37)^(35)^.

Achieving a reduction in Na intake, along with increasing K intake, is therefore an important goal which can have beneficial effects on population health. In 2013, the World Health Assembly agreed on a global target of a 30 % relative reduction in population Na/salt intake by 2025, as part of the Global Action Plan to reduce the growing burden of non-communicable diseases. This was to be achieved by comprehensive salt reduction strategies^(36)^. Such population-level reductions in Na/salt intake can be achieved through a combination of approaches, for example, consumer awareness campaigns or reformulation of processed foods and meals by the food manufacturers. In 2010, Slovenia accepted the National Action Plan for reducing salt intake, which among other activities included working with the food industry to gradually decrease salt content in processed foods (meat products, bread and bakery products, etc.), on a voluntary basis^(37)^. However, according to a recently published study, there were very limited effects of this food reformulation strategy on Na content in prepacked foods in the period between 2011 and 2020 in Slovenia^(38)^. Activities to reduce Na content and intake in the population should therefore strengthen if we wish to meet the global target of 30 % relative reduction in population Na intake.

### Strengths and limitations

Our study has several strengths. First, we collected data on Na and K intake using the gold standard method of 24-h urine collection. Second, we employed proper quality control of urine samples to minimise under- and over-collection. Third, we included the measurement of K excretion in 24-h urine, which was lacking in Slovenia, especially in adults. Fourth, previous estimates of salt intake in Slovenian adults were done on smaller sample sizes; therefore, our results better represent current salt intake in Slovenia.

There were also some limitations which should be considered. Despite multiple measures taken up to mitigate the non-response bias (e.g. multiple contacts with non-respondents and weighting of the data), it cannot be completely ruled out. Furthermore, we collected only one 24-h sample from each participant due to financial restraints and burdensome sample collection for both participants and researchers. Because of this, we cannot assess intake on an individual level due to high intra-individual variability. Despite instructing participants to eat normally during the collection period, it is also difficult to completely rule out bias.

## Conclusions

In conclusion, our study showed that estimated salt intake among adults in Slovenia remains high, despite relative improvement compared with previous studies. K intake among adults appears to be insufficient and should be improved. Both results are also reflected in the mean Na:K ratio, which was more than twice higher than the value that the WHO considers to be an optimal ratio. While our study indicated an improvement in salt intake among adults in Slovenia, activities to further reduce salt intake should strengthen, especially on Na content in prepacked foods and out-of-home meals.

## Supporting information

Kugler et al. supplementary material Kugler et al. supplementary material

## Supplementary material

For supplementary material accompanying this paper visit https://doi.org/10.1017/S1368980024001605
