# Supplementary material for: Twenty-four hour urinary sodium and potassium excretion in adult population of Slovenia: results of the Manjsoli.si/2022 study
Source: Public Health Nutr. 2024 Sep 16;27(1):e163. doi: 10.1017/S1368980024001605 (PMC11505472; doi:10.1017/S1368980024001605)
Supplement: Kugler et al. supplementary material [file S1368980024001605sup001.docx]

Supplementary Table 1: Comparisons of socio-demographic characteristics between the initial and final sample of the adult population of Slovenia.

|  | Initial sample | | Weighted data | | Unweighted data | |
| --- | --- | --- | --- | --- | --- | --- |
|  | n | % | n | % | n | % |
| Sex |  |  |  |  |  |  |
| - Male | 1047 | 52.4 | 270 | 52.1 | 227 | 43.8 |
| - Female | 953 | 47.7 | 248 | 47.9 | 291 | 56.2 |
| - Total | 2000 | 100 | 518 | 100 | 518 | 100 |
| Age |  |  |  |  |  |  |
| - 25-34 | 402 | 20.1 | 109 | 21.0 | 79 | 15.3 |
| - 35-44 | 533 | 26.7 | 138 | 26.7 | 102 | 19.7 |
| - 45-54 | 543 | 27.2 | 137 | 26.4 | 158 | 30.5 |
| - 55-64 | 522 | 26.1 | 134 | 25.9 | 179 | 34.6 |
| - Total | 2000 | 100 | 518 | 100 | 518 | 100 |
| Education |  |  |  |  |  |  |
| - Primary or less | 249 | 12.6 | 63 | 12.2 | 37 | 7.1 |
| - Secondary | 1133 | 57.5 | 285 | 55.1 | 288 | 55.6 |
| - Teritary or more | 587 | 29.8 | 169 | 32.7 | 193 | 37.3 |
| - Total | 1969 | 100 | 518 | 100 | 518 | 100 |
| Cohesion region |  |  |  |  |  |  |
| - West | 1070 | 53.5 | 274 | 52.9 | 274 | 52.9 |
| - East | 930 | 46.5 | 244 | 47.1 | 244 | 47.1 |
| - Total | 2000 | 100 | 518 | 100 | 518 | 100 |
| Type of settlement |  |  |  |  |  |  |
| - Rural settlements | 540 | 27.0 | 151 | 29.2 | 149 | 28.8 |
| - Smaller urban settlements | 500 | 25.0 | 122 | 23.5 | 127 | 24.5 |
| - Large urban settlements | 390 | 19.5 | 102 | 19.7 | 99 | 19.1 |
| - Cities with at least 10.000 inhabitants | 310 | 15.5 | 75 | 14.4 | 75 | 14.5 |
| - Ljubljana | 260 | 13.0 | 68 | 13.1 | 68 | 13.1 |
| - Total | 2000 | 100 | 518 | 100 | 518 | 100 |

Supplementary Table 2: Mean estimated salt intakes (g/day) by BMI categories in a sample of the adult population of Slovenia.

|  | Total  (n=518) | | Male  (n=227) | | Female  (n=291) | |
| --- | --- | --- | --- | --- | --- | --- |
|  | Mean | 95% CI | Mean | 95% CI | Mean | 95% CI |
| BMI categorization |  |  |  |  |  |  |
| - Underweight (<18 kg/m^2^ ) | 5.70 | (2.78-8.62) | 4.74 | (-28.9-38.3) | 6.19 | (2.00-10.4) |
| - Normal (18.5-24.9 kg/m^2^) | 8.41 | (7.85-8.95) | 9.49 | (8.46-10.5) | 7.72 | (7.12-8.32) |
| - Overweight (25-29.9 kg/m^2^) | 10.7^a^ | (9.93-11.4) | 11.2 | (10.3-12.1) | 9.81^a^ | (8.60-11.0) |
| - Obese (>30 kg/m^2^) | 11.7^a,b^ | (10.8-12.6) | 13.7^a,c^ | (12.5-14.9) | 8.65 | (7.82-9.48) |

BMI, body mass index; CI, Confidence interval. Significant differences between mean values within a column are depicted with superscript letter: ^a^ p<0.001 vs Normal; ^b^ p<0.05 vs Underweight; ^c^ p<0.01 vs Overweight. Results are based on two-sided tests assuming equal variances; tests are adjusted for all pairwise comparisons using the Bonferroni correction. p-value <0.05 was considered as statistically significant.

Supplementary Table 3: Mean estimated salt intakes (g/day) by blood pressure measurement categories in a sample of the adult population of Slovenia.

|  | Total  (n=518) | | Male  (n=227) | | Female  (n=291) | |
| --- | --- | --- | --- | --- | --- | --- |
|  | Mean | 95% CI | Mean | 95% CI | Mean | 95% CI |
| Blood pressure categorization |  |  |  |  |  |  |
| - <120 SBP and <80 DBP (mmHg) | 8.9 | (8.3-9.5) | 10.6 | (9.07-12.2) | 8.22 | (7.63-8.81) |
| - 120-129 SBP and/or 80-84 DBP (mmHg) | 9.8 | (9.0-10.5) | 10.8 | (9.86-11.8) | 7.96 | (6.89-9.03) |
| - 130-139 SBP and/or 85-89 DBP (mmHg) | 10.9^a^ | (9.7-12.0) | 12.0 | (10.5-13.5) | 8.54 | (7.23-9.85) |
| - 140-159 SBP and/or 90-99 DBP (mmHg) | 11.9^b,c^ | (10.7-13.0) | 12.7 | (11.4-13.9) | 10.4^a^ | (8.24-12.5) |
| - 160-179 SBP and/or100-109 DBP (mmHg) | 9.6 | (7.9-11.3) | 10.2 | (7.76-12.6) | 8.32 | (6.39-10.3) |
| - ≥180 SBP and/or ≥110 DBP (mmHg) | 11.6 | (0.3-22.9) | 12.9 | (-2.9-28.8) | 2.21 | / |

CI, Confidence interval; SBP, systolic blood pressure; DBP, diastolic blood pressure. Significant differences between mean values within a column are depicted with superscript letter: ^a^ p<0.05 vs <120 SBP and <80 DBP (mmHg); ^b^ p<0.001 vs <120 SBP and <80 DBP (mmHg); ^c^ p<0.05 vs 120-129 SBP and/or 80-84 DBP (mmHg). Results are based on two-sided tests assuming equal variances; tests are adjusted for all pairwise comparisons using the Bonferroni correction. p-value <0.05 was considered as statistically significant.
